# Time-restricted feeding restores metabolic flexibility in adult mice with excess adiposity

**DOI:** 10.3389/fnut.2024.1340735

**Published:** 2024-02-15

**Authors:** Lin Yan, Bret M. Rust, Daniel G. Palmer

**Affiliations:** United States Department of Agriculture, Agricultural Research Service, Grand Forks Human Nutrition Research Center, Grand Forks, ND, United States

**Keywords:** time-restricted feeding, diet, metabolomics, excess adiposity, mice

## Abstract

**Introduction:**

Obesity is prevalent with the adult population in the United States. Energy-dense diets and erratic eating behavior contribute to obesity. Time-restricted eating is a dietary strategy in humans that has been advanced to reduce the propensity for obesity. We hypothesized that time-restricted feeding (TRF) would improve metabolic flexibility and normalize metabolic function in adult mice with established excess adiposity.

**Methods:**

Male C57BL/6NHsd mice were initially fed a high-fat diet (HFD) for 12 weeks to establish excess body adiposity, while control mice were fed a normal diet. Then, the HFD-fed mice were assigned to two groups, either *ad libitum* HFD or TRF of the HFD in the dark phase (12 h) for another 12 weeks.

**Results and discussion:**

Energy intake and body fat mass were similar in TRF and HFD-fed mice. TRF restored rhythmic oscillations of respiratory exchange ratio (RER), which had been flattened by the HFD, with greater RER amplitude in the dark phase. Insulin sensitivity was improved and plasma cholesterol and hepatic triacylglycerol were decreased by TRF. When compared to HFD, TRF decreased transcription of circadian genes *Per1* and *Per2* and genes encoding lipid metabolism (*Acaca*, *Fads1, Fads2*, *Fasn*, *Scd1*, and *Srebf1*) in liver. Metabolomic analysis showed that TRF created a profile that was distinct from those of mice fed the control diet or HFD, particularly in altered amino acid profiles. These included aminoacyl-tRNA-biosynthesis, glutathione metabolism, and phenylalanine, tyrosine, and tryptophan biosynthesis pathways. In conclusion, TRF improved metabolic function in adult mice with excess adiposity. This improvement was not through a reduction in body fat mass but through the restoration of metabolic flexibility.

## Introduction

A 2017–2020 National Health and Nutrition Examination Survey found approximately 42% of adults are obese (1, 2) and nearly 31% of adults are overweight (3) in the United States. Obesity is a metabolic disorder whose occurrence is multifactorial. Modern lifestyle is an environmental contributor to obesity (e.g., energy dense diets and erratic eating behavior). Adults with obesity or excess adiposity have an increased risk for chronic diseases such as heart diseases, type 2 diabetes, and certain types of cancer. The estimated medical cost of obesity in the United States was nearly 173 billion in 2019 (4). Medical costs for adults who were obese were $1,861 greater than those with healthy body weight (4).

Human studies have shown that metabolic syndrome (5, 6), unhealthy food intake and weight gain (7), and obesity (7, 8) are associated with night-shift workers, which indicates an effect on biological clocks that exist in all human organs. These clocks cycle every 24 h in a diurnal pattern and control the daily rhythms of human life (e.g., eating vs. fasting; sleep vs. wakefulness), which are essential for health and wellbeing. Disruption of biological clocks alters rhythms of daily life and metabolic flexibility (ability of the body to alter metabolism in response to available fuel), and thus it increases the risk for metabolic disorders and chronic diseases.

Laboratory studies with rodents have found that disruption of circadian rhythms affects metabolic flexibility. Food intake increased in the light phase (the rest phase for nocturnal rodents) and weight gain occurred when mice were fed a high-fat diet (9). Energy-dense, high-fat diets disturbed daily oscillations of respiratory exchange ratio (10, 11) and altered circadian rhythms (11–13) in mice when compared to those fed a healthy control diet.

Time-restricted eating is a dietary strategy in humans that has been advanced for reducing the risk for obesity. It makes food available at a fixed time of the day so that the daily feeding and fasting cycle aligns with circadian clocks. Short-term time-restricted eating interventions reduces energy intake and decreases body weight or body fat mass in healthy human subjects (14–16) and in subjects who are overweight or obese (17, 18). In animal studies, initiation of time-restricted feeding (TRF) prior to the induction of excess fat mass restores metabolic rhythms, improves insulin sensitivity, and reduces body weight in mice fed a high-fat diet (10, 11, 19). Greater protection occurs when TRF is initiated at a younger age (10, 19). For the present study, we hypothesized that TRF would improve metabolic profiles in adult mice with established excess adiposity. To test this hypothesis, the effect of TRF of a high-fat diet during the dark phase (the active phase for nocturnal rodents) on untargeted metabolomics of primary metabolism was determined in adult mice with excess body fat mass.

## Materials and methods

### Animals and diets

C57BL/6NHsd mice (male, 3 to 4-week-old) were purchased from Envigo, Madison, WI. Mice were maintained in a pathogen-free room (temperature 22 ± 1°C) on a 12:12-h light/dark cycle. Diets were prepared according to a modified AIN93G formulation (20) that provided 16 and 48% of energy from dietary oils (referred to as control and high-fat diet, HFD), respectively (Table 1). Both diets (powder diets) were stored at-20°C. Fresh diets were provided to mice every other day. Mice had free access to their diets and deionized drinking water. They were weighed weekly.

**Table 1:** Diet composition.

Ingredient	Control g/kg	High-fat g/kg
Casein	200	250
Cornstarch	540	250
Sucrose	100	125
Cellulose	41	62
Fat	71	266
Palm oil	20.9	137.1
Cocoa butter	12.1	90.4
Flaxseed oil	8.5	10.5
Safflower oil	26.8	17.3
Coconut oil	2.8	10.6
L-Cystine	3	3.6
Vitamin mix	10	12
Mineral mix	35	42
Choline bitartrate	3	3
t-Butylhydroquinone	0.014	0.02
Total	1003	1013.6
Energy, %		
Protein	20	20
Carbohydrates	64	32
Fat	16	48
Gross energy, kcal/g	4.08	4.99

Palm oil (no. 18928), cocoa butter (no. 80612), and linoleic safflower oil (no. 5004) were obtained from Natural Oils International, Inc. (Simi Valley, CA, United States). Flaxseed oil (no. 20123) was from Dyets, Inc. (Bethlehem, PA, United States).

### Experimental design

Body weight was used as the primary endpoint for power analysis. Using a one-way ANOVA, 11 mice per group were required to be able to achieve 90% power to detect a 15% reduction in body weight of the group with the restricted feeding, given a within-group standard deviation of 3.4 and α = 0.05. One mouse was added to each group to account for attrition for a total of 12 mice per group.

This study was conducted in two phases. Phase one was designed to induce excess body adiposity. Phase two investigated the effect of imposing TRF on metabolic changes caused by excess adiposity. Mice were acclimated to the control diet for one week. Then, they were assigned randomly into two groups and fed the control diet (*n* = 24) and HFD (*n* = 36), respectively. In phase one, 12 mice from each group were terminated after 11 weeks of feeding the HFD. In phase two, the remaining mice were maintained on their respective diets, except half of the HFD-fed mice (*n* = 12) were assigned to TRF at the end of the 12th week of phase one. The duration of phase two was 12 weeks. Mice assigned to the TRF group had free access to the HFD in the dark phase (Zeitgeber time, ZT, 12–24). Zeitgeber time 0 and 12 are the times at which the light is on and off, respectively. Food availability to the TRF group was regulated by transferring mice daily between cages with water only (ZT 0–12) and cages with both diet and water (ZT 12–24). Food intake was recorded seven days per week for three consecutive weeks starting from the fourth week of the HFD in phase one and the fourth week of TRF in phase two. One week after the food intake measurements, body composition of each mouse was evaluated for both phase one and phase two by using an Echo whole-body composition analyzer (Model 100, Echo Medical System, Houston, TX, United States). At the end of the study, mice were injected intraperitoneally with a mixture of ketamine and xylazine followed by exsanguination at ZT 5–7. Livers were snap-frozen in liquid nitrogen and plasma was collected by using EDTA (4.5%) rinsed syringes. Livers and plasma were stored at-80°C until they were analyzed.

### Whole body metabolism determination

Whole body metabolism was assessed on the eighth week of phase one and the eighth week of phase two by using indirect calorimetry (Comprehensive Laboratory Animal Monitoring System, Columbus Instruments, Columbus, OH, United States). Mice were acclimated in metabolic chambers for 24 h before their rates of O_2_ consumption (VO_2_) and CO_2_ production (VCO_2_) were recorded for 1 min every 12 min over a 48-h period. The respiratory exchange ratio (RER) was calculated for both light and dark phases by using the formula RER = VCO_2_/VO_2_.

### Quantification of blood glucose and plasma metabolic biomarkers

Blood glucose was quantified by using an Accu-Chek Aviva blood glucose meter (Roche Diagnostics, Indianapolis, IN, United States) before the anaesthetization. Sandwich enzyme-linked immunosorbent assay kits were used to quantify insulin (Mercodia, Inc., Winston Salem, NC, United States), leptin, and adiponectin (R&D Systems, Minneapolis, MN, United States) in plasma. Homeostatic model assessment of insulin resistance (HOMA-IR) was calculated by using the formula HOMA-IR = (glucose x insulin)/ 405 with glucose expressed as mg/dL and insulin as mU/L.

### Quantification of lipids in plasma and liver

Plasma cholesterol (analyzer no. 03039773) and HDL-cholesterol (analyzer no. 07528566) and hepatic triacylglycerol (analyzer no. 20767107) were determined by using a COBAS INTEGRA 400 Plus analyzer (Roche Diagnostics, Ramsey, MN, United States).

### Metabolomic analysis

Metabolomic analysis of liver samples from phase two (*n* = 12 per group) was performed at the West Coast Metabolomics Center (University of California-Davis, Davis, CA, United States) (21, 22). Liver samples were extracted by the acetonitrile/isopropanol/H_2_O (3:3:2) buffer, resuspended in a diluted acetonitrile solution (acetonitrile/H_2_O, 1:1), and analyzed by gas chromatography time-of-flight mass spectrometry (GC-TOF-MS) for untargeted metabolomics of primary metabolism. The BinBase database was used to process obtained data (23). Quantifier ion peak heights were normalized to the sum intensities of all known compounds identified. Compounds constituting ≤0.02% of total signal intensity were excluded from the analysis. Moreover, compounds that could not be identified as metabolites or intermediates common to mammalian metabolism according to the Kyoto Encyclopedia of Genes and Genomes (KEGG) Database or the Human Metabolome database (24–26) were excluded from the analysis.

### RNA isolation and real-time quantitative PCR

Liver samples from phase two (*n* = 12 per group) were isolated for total RNA by using the RNeasy Lipid Tissue Mini Kit (Qiagen, Germantown, MD, United States). Isolated RNA was analyzed for purity by using a NanoDrop 8,000 Spectrophotometer (Thermo Scientific, Wilmington, DE, United States). cDNA was synthesized by using the high-capacity cDNA reverse transcription kit (Applied Biosystems, Waltham, MA, United States). Real-time quantitative PCR of circadian locomotor output cycles kaput (*Clock*) (Mm00455950_ml), aryl hydrocarbon receptor nuclear translocator-like protein 1 (*Bmal1*/*Arntl*) (Mm00500223_ml), period-1 (*Per1*) and *Per2* (Mm00501813_ml and Mm00478099_ml), cryptochrome 1 (*Cry1*) (Mm00514392_ml), nuclear receptor subfamily 1 group D member 1 (*Nr1d1/Rev-erb*) (Mm00520708_ml), acetyl-CoA carboxylase (*Acaca*) (Mm01304257_m1), fatty acid desaturase (*Fads*) *1* and *2* (Mm00507605_m1 and Mm00517221_m1), fatty acid synthase (*Fasn*) (Mm00662319_m1), stearoyl-CoA desaturase 1 (*Scd1*) (Mm00772290_m1), and sterol regulatory element-binding protein 1 (*Srebf1*) (Mm00550338_m1) was determined and normalized to 18 s rRNA by using the TaqMan Assay of Demand primers on ABI QuantStudio 12 K-flex real-time quantitative PCR system (Applied Biosystems). Probes for the transcripts analyzed and 18 s rRNA were obtained from Applied Biosystems. The 2^-ΔΔCT^ method was used to calculate the relative changes in gene transcription (27).

### Statistical analyses

Differences in weekly body weight between groups were analyzed by using a mixed model (repeated measures ANOVA). Bonferroni corrections were applied across all comparisons between control and HFD groups at each week for phase one. Tukey’s Honestly Significant Difference test corrections were used for all pairwise comparisons among the control, HFD, and TRF groups at each week for phase two. One-way ANOVA followed by Tukey contrasts were performed to compare differences in metabolite analyses among the control, HFD, and TRF groups with false discovery rate (FDR) -adjusted *p* values reported (SAS 9.4, SAS Institute, Cary, NC, United States). A mixed model with fixed effects of diet, light/dark phases, its interaction, and a random effect of mouse was performed to compare differences in RER among the three groups with Bonferroni-adjusted *p* values reported. Metabolomic data were normalized by the Pareto scaling method and analyzed by using sparse partial least square-discriminant analysis (sPLS-DA) (MetaboAnalyst 5.0, McGill University, Sainte Anne de Bellevue, Quebec, Canada) (28, 29). Functional relationships of identified metabolites between treatment groups (control vs. HFD, HFD vs. TRF, and control vs. TRF) were analyzed and mapped by using the KEGG global metabolic network analysis and the metabolite-metabolite interaction network analysis (MetaboAnalyst 5.0). Obtained results from treatment groups are presented as fold changes to the control group. Results (means ± standard error of the mean, SEM) with differences at *p* ≤ 0.05 are considered significant.

## Results

### Findings from phase one

#### Body weight, body composition, and whole-body metabolism

**Figure 1:**
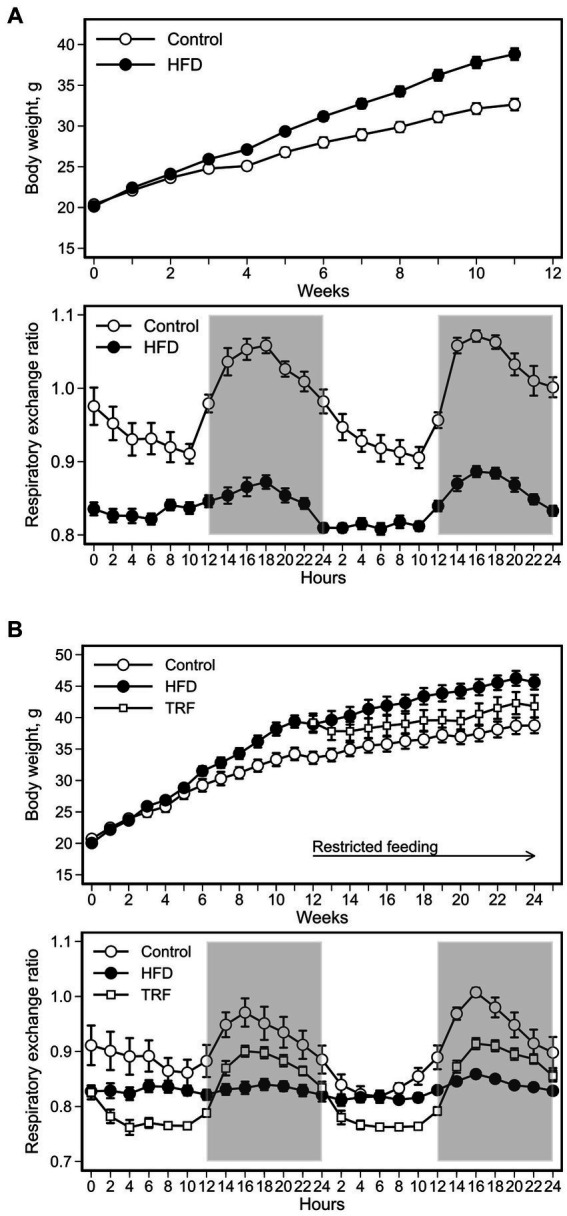
Body weight and respiratory exchange ratio (RER) of mice fed the control diet or high-fat diet (HFD) for phase one **(A)** and mice fed the control diet, HFD, or time-restricted feeding (TRF) of the HFD for phase two **(B)**. In phase one, the HFD increased body weight compared to the control diet; the difference became significant after five weeks on the HFD (*p* ≤ 0.05). In phase two, TRF decreased body weight, but the difference was not significant from that with nonrestricted feeding of the HFD for the duration of the study. Values are means ± SEM (body weight: *n* = 12 per group for both phases; RER: *n* = 12 per group for phase one, *n* = 9 per group for phase two). Open space = light phase, gray space = dark phase.

The HFD increased body weight compared to the control diet (Figure 1A). The difference became significant five weeks after initiating the HFD (*p* = 0.05) and remained significant for the remainder of phase one (Figure 1A). The final weight gain at the end of this phase was 11.0 ± 0.9 g and 16.7 ± 1.6 g for the control and HFD groups, respectively (*p* = 0.01).

**Figure 2:**
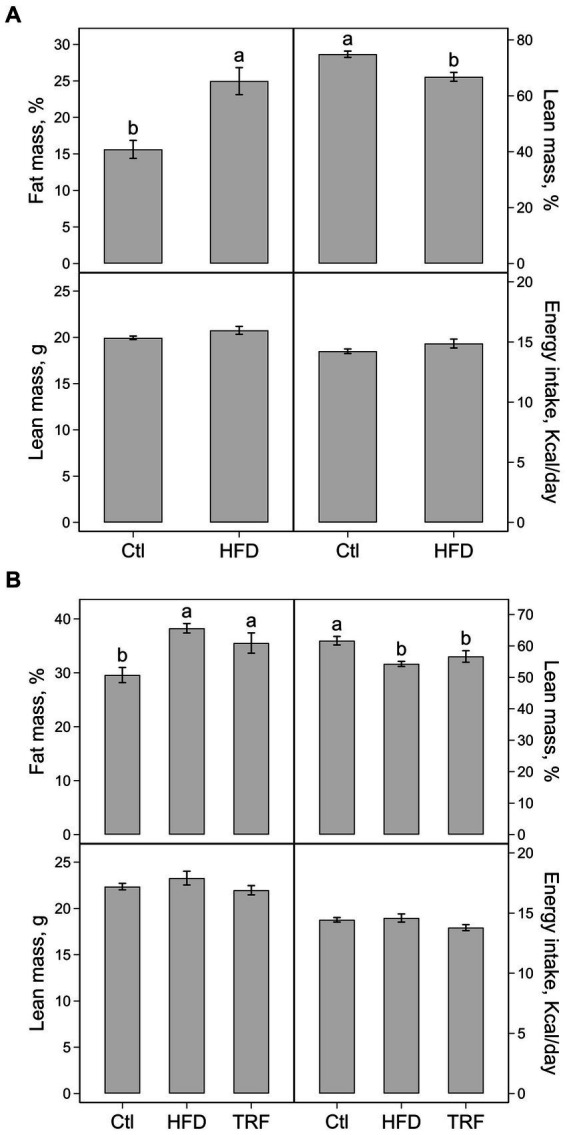
Percentage of body fat mass, percentage of body lean mass, absolute lean mass, and energy intake of mice fed the control diet (Ctl) or high-fat diet (HFD) for phase one **(A)** and mice fed the Ctl, HFD, or time-restricted feeding (TRF) of the HFD for phase two **(B)**. Values (means ± SEM) in each panel with different letters are significant at *p* ≤ 0.05 (*n* = 12 per group).

The percent body fat mass was 15.6 and 25.0% (*p* < 0.01) and percent body lean mass was 74.9 and 66.8% (*p* < 0.01) for the control and HFD groups, respectively (Figure 2A). Significant differences did not occur between the control and HFD groups in absolute body lean mass (20.0 ± 0.2 vs. 21.0 ± 0.4 g) and energy intake (14.2 ± 0.2 vs. 14.9 ± 0.4 kcal/day) (Figure 2A).

RER of control mice oscillated in a diurnal pattern peaking in the dark phase (Figure 1A). RER oscillations significantly diminished in HFD-fed mice compared to control mice (Figure 1A; Table 2). Greater RER occurred in the dark phase than in the light phase for both control and HFD groups (Table 2).

**Table 2:** Respiratory exchange ratio (RER) of mice with different dietary treatments in phases one and two.

Phase 1	Control	Hight-fat
RER, mean	0.97 ± 0.01^a^	0.84 ± 0.01^b^
RER, light phase	0.91 ± 0.01^a^	0.81 ± 0.01^b^
RER, dark phase	1.02 ± 0.01^a*^	0.86 ± 0.01^b*^

Values (means ± SEM) with different letters in the same row are different at *p* ≤ 0.05; values in the same column with a * are different at *p* ≤ 0.05 when dark phase is compared to light phase (Bonferroni-adjusted *p* values). *N* = 12 per group for phase 1, *n* = 9 per group for phase 2. TRF: time-restricted feeding.

#### Metabolic biomarkers in plasma and liver

When compared to the control diet, the HFD increased concentrations of glucose in blood (15.5%) and insulin in plasma (120%) and calculated HOMA-IR (155%) (Table 3). The plasma concentration of leptin was 118% higher in HFD-fed mice than in control mice; adiponectin did not differ between the two groups (Table 3). The HFD increased plasma concentrations of total cholesterol and HDL-cholesterol each by 15% over that of the control diet (Table 3). Hepatic triacylglycerol content of the HFD group was 105% greater than that of the control group (Table 3).

**Table 3:** Metabolic biomarkers in plasma and triacylglycerol in liver from mice with different dietary treatments in phases one and two.

Phase 1	Control	High-fat
Plasma		
Glucose (blood), mg/dL	138.96 ± 4.47^b^	160.46 ± 8.73^a^
Insulin, mU/L	11.71 ± 1.86^b^	25.76 ± 2.99^a^
HOMA-IR	4.12 ± 0.70^b^	10.51 ± 19.18^a^
Leptin, ng/mL	14.88 ± 0.68^b^	32.38 ± 5.36^a^
Adiponectin, μg/mL	6.82 ± 0.18	6.61 ± 0.15
Total cholesterol, mg/dL	92.42 ± 2.46^b^	106.19 ± 4.48^a^
HDL cholesterol, mg/dL	81.28 ± 2.24^b^	93.42 ± 3.15^a^
Liver		
Triacylglycerol, mg/g	18.62 ± 2.66^b^	38.11 ± 5.04^a^

Values (means ± SEM) in the same row with different letters are significant at *p* ≤ 0.05 (*n* = 12 per group). HOMA-IR: Homeostatic model assessment of insulin resistance, HDL: high-density lipoprotein, TRF: time-restricted feeding.

### Findings from phase two

#### Body weight, body composition, and whole-body metabolism

At the start of phase two, body weights were 33.4 ± 1.0 g for control mice (*n* = 12) and 39.1 ± 1.0 g for HFD-fed mice (*n* = 24) (*p* < 0.01, Figure 1B). Body weight of TRF mice was non-significantly lower than that of HFD-fed mice throughout this phase (Figure 1B). Compared to the start, at the end of phase two body weight gain was notably lower in TRF mice (2.7 ± 0.7 g) than in HFD-fed mice (6.5 ± 1.2 g) (*p* = 0.01). Weight gain did not differ between the HFD-fed mice and control mice (5.4 ± 0.6 g) in this phase.

The percent body fat mass was 29.6 ± 1.4%, 38.3 ± 0.9%, and 35.5 ± 1.9% and percent body lean lass was 61.7 ± 1.4%, 54.3 ± 0.8%, and 56.6 ± 1.8% for the control, HFD, and TRF groups, respectively (Figure 2B). The percent body fat mass and percent lean mass in both HFD-fed and TRF mice differed from control mice (*p* ≤ 0.05) but were not different from each other. The absolute lean mass and energy intake were similar among the three groups. Absolute lean masses were 22.4 ± 0.4, 23.3 ± 0.7, and 22.0 ± 0.5 g and energy intakes were 14.4 ± 0.2, 14.6 ± 0.3 and 13.8 ± 0.3 kcal/day for the control, HFD, and TRF groups, respectively (Figure 2B).

The HFD flattened RER oscillations shown by the control group; TRF restored the dampened oscillations (Figure 1B). When the light and dark phases were compared separately, RER oscillations in the light phase of the TRF group was lower than those in the control and HFD groups (Table 2). However, dark phase RER oscillations increased from the light phase similarly in both TRF and control groups, but not in the HFD group (Table 2).

#### Metabolic biomarkers in plasma and liver

Blood glucose concentration of the HFD group was 12% higher than that of the control group; there was no difference in blood glucose between the HFD and TRF groups (Table 3). The HFD increased plasma concentrations of insulin (202%) and leptin (58%) compared those found with the control group; TRF decreased insulin (77%) and leptin (39%) compared to the HFD (Table 3). Similar changes occurred in HOMA-IR. Feeding the HFD instead of the control diet decreased plasma adiponectin concentration by 19%. TRF increased adiponectin by 14% compared to the HFD (*p* = 0.01, Table 3).

Compared to the control diet, the HFD increased plasma concentrations of total cholesterol (33.8%) and HDL cholesterol (26%); TRF decreased total cholesterol (22%) and HDL cholesterol (17%) compared to the HFD (Table 3). Hepatic triacylglycerol content of HFD-fed mice was 290% higher than that of control mice. TRF resulted in a 49% decrease in hepatic triacylglycerol compared to the HFD (Table 3).

#### Hepatic transcription of circadian genes and genes encoding lipid metabolism

**Figure 3:**
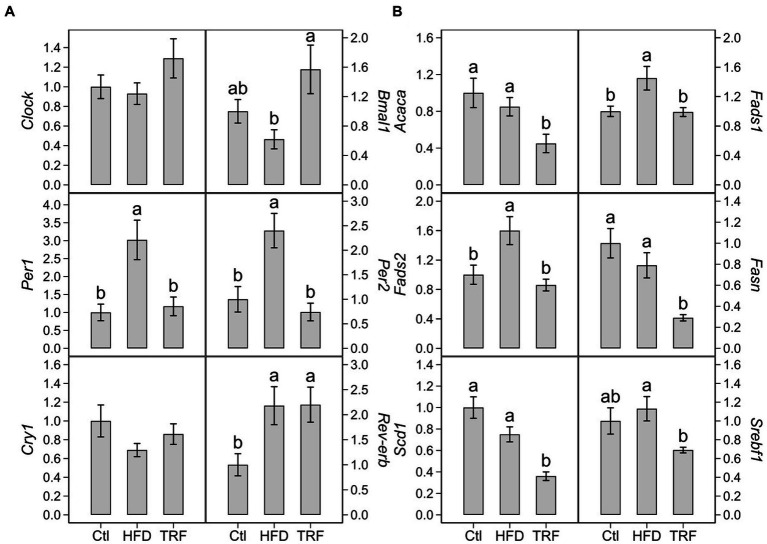
Hepatic transcription of circadian genes **(A)** and genes encoding lipid metabolism **(B)** in mice fed the control diet (Ctl), high-fat diet (HFD), or time-restricted feeding (TRF) of the HFD. Values are means ± SEM (*n* = 12 per group). Comparisons in each panel with different letters are significant at *p* ≤ 0.05.

The HFD increased transcription of *Per1* (200%), *Per2* (140%), *Rev-erb* (118%) over that of the control diet (Figure 3A; Supplementary Table S1). TRF decreased transcription of *Per1* (60%) and *Per2* (70%) compared to the HFD but did not change *Rev-erb* (Figure 3A). TRF elevated *Bmal1* transcription (153%) compared to the HFD, which was similar to that found in the control group (Figure 3A). There were no differences in *Clock* and *Cry1* transcription among the three groups (Figure 3A).

Compared to the control diet, the HFD elevated transcription of *Fads1* (45%) and *Fads2* (60%) (Figure 3B; Supplementary Table S1). There were no significant differences in transcription of *Acaca*, *Fasn*, *Scd1*, and *Srebf1* between the control and HFD groups (Figure 3B). TRF decreased transcription of *Acaca* (47%), *Fads1* (32%), *Fads2* (46%), *Fasn* (63%), *Scd1* (47%), and *Srebf1* (39%) compared to the HFD (Figure 3B).

#### Hepatic metabolomes

We identified 160 compounds from 529 discrete signals (Supplementary Table S2). Among them, 87 met the criteria for metabolomic and statistical analyses. These 87 metabolites were grouped into four categories related to amino acid, energy, lipid, and nucleotide and vitamin metabolism (Tables 4–7).

**Table 4:** Identified metabolites related to amino acid metabolism in liver from mice fed the control diet, high-fat diet, or time-restricted feeding (TRF) of the high-fat diet.

	Control	High-fat	TRF
Alanine	1 ± 0.05	0.78 ± 0.09	0.78 ± 0.06
Aminomalonic acid	1 ± 0.13	1.04 ± 0.10	1.01 ± 0.10
Asparagine	1 ± 0.08^a^	0.85 ± 0.06^ab^	0.65 ± 0.05^b^
Aspartic acid	1 ± 0.18	1.41 ± 0.21	1.50 ± 0.12
β-alanine	1 ± 0.11	0.99 ± 0.12	0.72 ± 0.05
Creatinine	1 ± 0.16	1.12 ± 0.15	1.14 ± 0.15
Cysteine-glycine	1 ± 0.12^a^	0.62 ± 0.06^b^	0.64 ± 0.06^b^
Glutamic acid	1 ± 0.14	1.35 ± 0.08	1.22 ± 0.10
Glutamine	1 ± 0.14	1.21 ± 0.11	1.30 ± 0.09
Glutathione	1 ± 0.04	0.72 ± 0.11	1.00 ± 0.08
Glycine	1 ± 0.05	0.85 ± 0.05	1.02 ± 0.06
Glycyl-glycine	1 ± 0.04	0.99 ± 0.03	0.92 ± 0.04
Hypotaurine	1 ± 0.13^a^	0.80 ± 0.12^ab^	0.45 ± 0.05^b^
Isoleucine	1 ± 0.06	1.03 ± 0.06	0.99 ± 0.08
Leucine	1 ± 0.06^a^	0.87 ± 0.04^ab^	0.73 ± 0.04^b^
Lysine	1 ± 0.06	0.92 ± 0.08	0.78 ± 0.04
Methionine	1 ± 0.08^a^	0.69 ± 0.06^b^	0.50 ± 0.05^b^
Ornithine	1 ± 0.05^a^	0.89 ± 0.10^a^	0.59 ± 0.04^b^
Oxoproline	1 ± 0.05^b^	1.13 ± 0.05^ab^	1.29 ± 0.05^a^
Phenylalanine	1 ± 0.08^a^	0.89 ± 0.08^a^	0.61 ± 0.05^b^
Proline	1 ± 0.12^a^	0.67 ±0.07^b^	0.54 ± 0.07^b^
Serine	1 ± 0.09	0.93 ± 0.05	0.80 ± 0.06
Taurine	1 ± 0.14	0.85 ± 0.19	0.85 ± 0.11
Threonine	1 ± 0.07^a^	0.83 ± 0.05^ab^	0.65 ± 0.05^b^
Tryptophan	1 ± 0.03	0.91 ± 0.06	0.84 ± 0.05
Tyrosine	1 ± 0.04^a^	0.91 ± 0.05^a^	0.67 ± 0.03^b^
Urea	1 ± 0.04	1.00 ± 0.08	0.89 ± 0.08
Valine	1 ± 0.09	0.85 ± 0.04	0.76 ± 0.10
2-picolinic acid	1 ± 0.07	1.19 ± 0.09	1.25 ± 0.12

Values of treatment groups are normalized to that of the control group (*n* = 12 per group). Values (means ± SEM) in the same row with different letters are significant at *p* ≤ 0.05 (FDR-adjusted *p* values).

**Table 5:** Identified metabolites related to energy metabolism in liver from mice fed the control diet, high-fat diet, or time-restricted feeding (TRF) of the high-fat diet.

	Control	High-fat	TRF
Citric acid	1 ± 0.18^ab^	1.09 ± 0.18^a^	0.51 ± 0.09^b^
Fructose	1 ± 0.15^a^	0.59 ± 0.14^ab^	0.34 ± 0.06^b^
Fructose-1-phosphate	1 ± 0.16^a^	0.59 ± 0.14^ab^	0.36 ± 0.06^b^
Fructose-6-phosphate	1 ± 0.14	1.10 ± 0.30	1.09 ± 0.22
Fumaric acid	1 ± 0.15^b^	2.80 ± 0.47^a^	3.98 ± 0.40^a^
Glucose	1 ± 0.06	0.93 ± 0.09	1.18 ± 0.08
Glucose-1-phosphate	1 ± 0.16	1.00 ± 0.24	0.70 ± 0.12
Glucose-6-phosphate	1 ± 0.04^a^	0.81 ± 0.05^b^	0.76 ± 0.05^b^
Glucuronic acid	1 ± 0.05^b^	1.33 ± 0.10^a^	1.34 ± 0.07^a^
Glycerol-α-phosphate	1 ± 0.20^b^	1.88 ± 0.28^b^	3.41 ± 0.54^a^
Lactic acid	1 ± 0.04	0.88 ± 0.09	0.70 ± 0.08
Malic acid	1 ± 0.16^b^	2.91 ± 0.50^a^	3.18 ± 0.34^a^
Maltose	1 ± 0.08	0.82 ± 0.09	0.66 ± 0.14
Maltotriose	1 ± 0.08^a^	0.78 ± 0.10^ab^	0.53 ± 0.07^b^
Mannose	1 ± 0.06^a^	0.70 ± 0.09^b^	0.46 ± 0.07^b^
Myoinositol	1 ± 0.05	1.14 ± 0.09	1.24 ± 0.09
Sorbitol	1 ± 0.11^a^	0.60 ± 0.10^b^	0.39 ± 0.04^b^
Succinic acid	1 ± 0.15^b^	1.43 ± 0.15^b^	6.18 ± 1.96^a^
Succinic acid	1 ± 0.15^b^	1.43 ± 0.15^b^	4.50 ± 1.09^a^

Values of treatment groups are normalized to that of the control group (*n* = 12 per group). Values (means ± SEM) in the same row with different letters are significant at *p* ≤ 0.05 (FDR-adjusted *p* values).

**Table 6:** Identified metabolites related to lipid metabolism in liver from mice fed the control diet, high-fat diet, or time-restricted feeding (TRF) of the high-fat diet.

	Control	High-fat	TRF
Arachidic acid	1 ± 0.04^b^	1.00 ± 0.08^b^	1.56 ± 0.23^a^
Arachidonic acid	1 ± 0.05^b^	1.30 ± 0.06^a^	1.13 ± 0.09^ab^
Behenic acid	1 ± 0.05	0.98 ± 0.07	1.21 ± 0.10
Caproic acid	1 ± 0.08	1.18 ± 0.23	1.29 ± 0.14
Cholesterol	1 ± 0.04^b^	1.35 ± 0.07^a^	1.35 ± 0.04^a^
Dihydrocholesterol	1 ± 0.12	1.19 ± 0.17	1.14 ± 0.10
Ethanolamine	1 ± 0.20^b^	18.05 ± 5.16^ab^	24.01 ± 6.99^a^
Glycerol	1 ± 0.06	1.07 ± 0.09	0.76 ± 0.14
Heptadecanoic acid	1 ± 0.06	1.01 ± 0.13	1.28 ± 0.12
Inositol-4-monophosphate	1 ± 0.16^b^	1.60 ± 0.14^ab^	1.94 ± 0.19^a^
Linoleic acid	1 ± 0.06	0.80 ± 0.07	0.83 ± 0.12
Myristic acid	1 ± 0.10	0.99 ± 0.12	1.33 ± 0.14
Oleic acid	1 ± 0.06	1.13 ± 0.11	1.16 ± 0.26
Palmitoleic acid	1 ± 0.07^a^	0.55 ± 0.04^b^	0.63 ± 0.16^ab^
Phosphate	1 ± 0.04^b^	1.18 ± 0.05^a^	1.23 ± 0.05^a^
Phosphoethanolamine	1 ± 0.09	1.54 ± 0.19	1.52 ± 0.18
Squalene	1 ± 0.16^b^	2.88 ± 0.38^a^	1.82 ± 0.25^b^
Stearic acid	1 ± 0.09	1.06 ± 0.16	1.24 ± 0.14
Undecanoic acid	1 ± 0.09	1.30 ± 0.19	4.75 ± 2.18
1-monopalmitin	1 ± 0.09	1.20 ± 0.19	1.34 ± 0.17
4-hydroxybutyric acid	1 ± 0.12	1.22 ± 0.13	1.09 ± 0.17

Values of treatment groups are normalized to that of the control group (*n* = 12 per group). Values (means ± SEM) in the same row with different letters are significant at *p* ≤ 0.05 (FDR-adjusted *p* values).

**Table 7:** Identified metabolites related to nucleotide and vitamin metabolism in liver from mice fed the control diet, high-fat diet, or time-restricted feeding (TRF) of the high-fat diet.

	Control	High-fat	TRF
Nucleotides			
Adenine	1 ± 0.05	1.13 ± 0.10	1.23 ± 0.06
Adenosine	1 ± 0.09^b^	1.21 ± 0.11^b^	2.02 ± 0.18^a^
Adenosine-5-monophosphate	1 ± 0.20	1.60 ± 0.36	1.79 ± 0.43
Hypoxanthine	1 ± 0.19	0.85 ± 0.18	0.56 ± 0.10
Inosine	1 ± 0.10^a^	0.79 ± 0.11^ab^	0.56 ± 0.06^b^
Inosine 5-monophosphate	1 ± 0.21	1.28 ± 0.24	1.47 ± 0.20
N-methyl-UMP	1 ± 0.14^b^	1.76 ±0.25^ab^	1.93 ± 0.22^a^
Pyrophosphate	1 ± 0.10^b^	1.31 ± 0.18^ab^	1.87 ± 0.20^a^
Ribose	1 ± 0.11^ab^	1.11 ± 0.18^a^	0.57 ± 0.13^b^
UDP-N-acetylglucosamine	1 ± 0.07	1.09 ± 0.09	1.22 ± 0.12
Uracil	1 ± 0.09	1.38 ± 0.26	0.85 ± 0.13
Uridine	1 ± 0.07	0.89 ± 0.12	0.67 ± 0.08
Xanthine	1 ± 0.06^a^	0.87 ± 0.09^a^	0.56 ± 0.08^b^
5-methylthioadenosine	1 ± 0.04^b^	1.45 ± 0.11^a^	1.25 ± 0.07^ab^
Vitamins			
Ascorbic acid	1 ± 0.38	0.52 ± 0.05	0.62 ± 0.04
Dehydroascorbic acid	1 ± 0.06^b^	1.16 ± 0.07^b^	1.44 ± 0.04^a^
Flavin adenine	1 ± 0.08^ab^	1.13 ± 0.09^a^	0.71 ± 0.08^b^
Nicotinamide	1 ± 0.03	0.98 ± 0.07	0.82 ± 0.09
α-Tocopherol	1 ± 0.12^b^	1.60 ± 0.18^ab^	1.77 ± 0.21^a^

Values of treatment groups are normalized to that of the control group (*n* = 12 per group). Values (means ± SEM) in the same row with different letters are significant at *p* ≤ 0.05 (FDR-adjusted *p* values).

**Figure 4:**
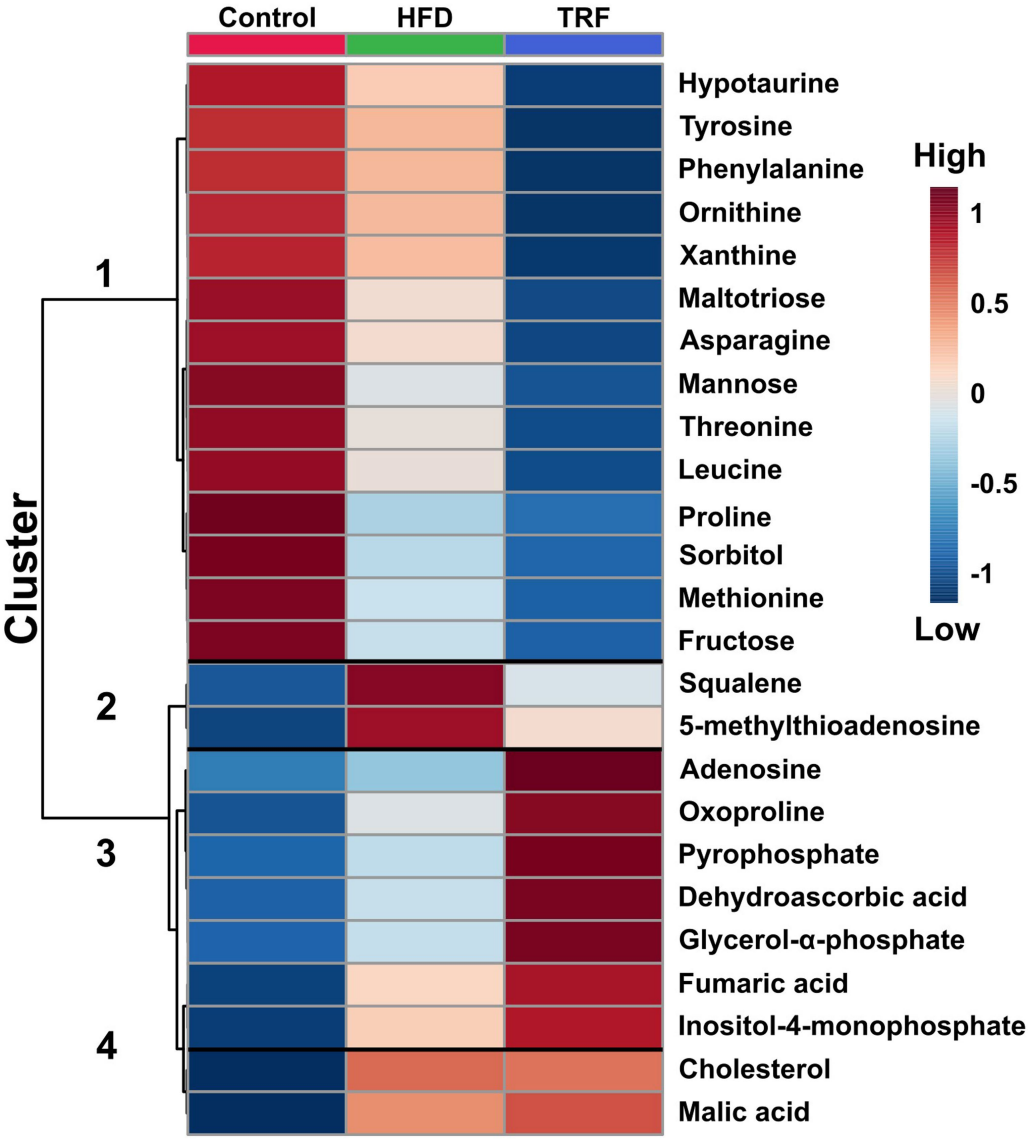
Hierarchical clustering heatmap of the 25 metabolites in liver that differed most significantly among the control, high-fat diet (HFD), and time-restricted feeding (TRF) of the HFD groups (FDR-adjusted *p* ≤ 0.05). Each square represents a mean of 12 samples. The dark-red color indicates greater signal strength; the blue color indicates weaker signal strength by GC-TOF-MS.

Heatmap analysis categorized 25 metabolites that were most significant among the three groups into four clusters (Figure 4). Cluster one contained 14 metabolites with signals that were lower in the TRF group than in the control and HFD groups (Figure 4). Nine in cluster one were amino acids and their metabolites; these were hypotaurine, tyrosine, phenylalanine, ornithine, asparagine, threonine, leucine, proline, and methionine. The other metabolites were related to energy metabolism (maltotiose, mannose, sorbitol, and fructose) and the nucleotide metabolite xanthine. In cluster two, squalene and 5-methylthioadenosine were distinctly elevated in the HFD group compared to the control group, but these elevations did not occur in the TRF group. Cluster three contained seven metabolites (adenosine, oxoproline, pyrophosphate, dehydroascorbic acid, glycerol-α-phosphate, fumaric acid, and inositol-4-monophosphate) whose signals were higher in the TRF group than in both control and HFD groups. In cluster four, cholesterol and malic acid signals were greater in both HFD and TRF groups than in the control group.

**Figure 5:**
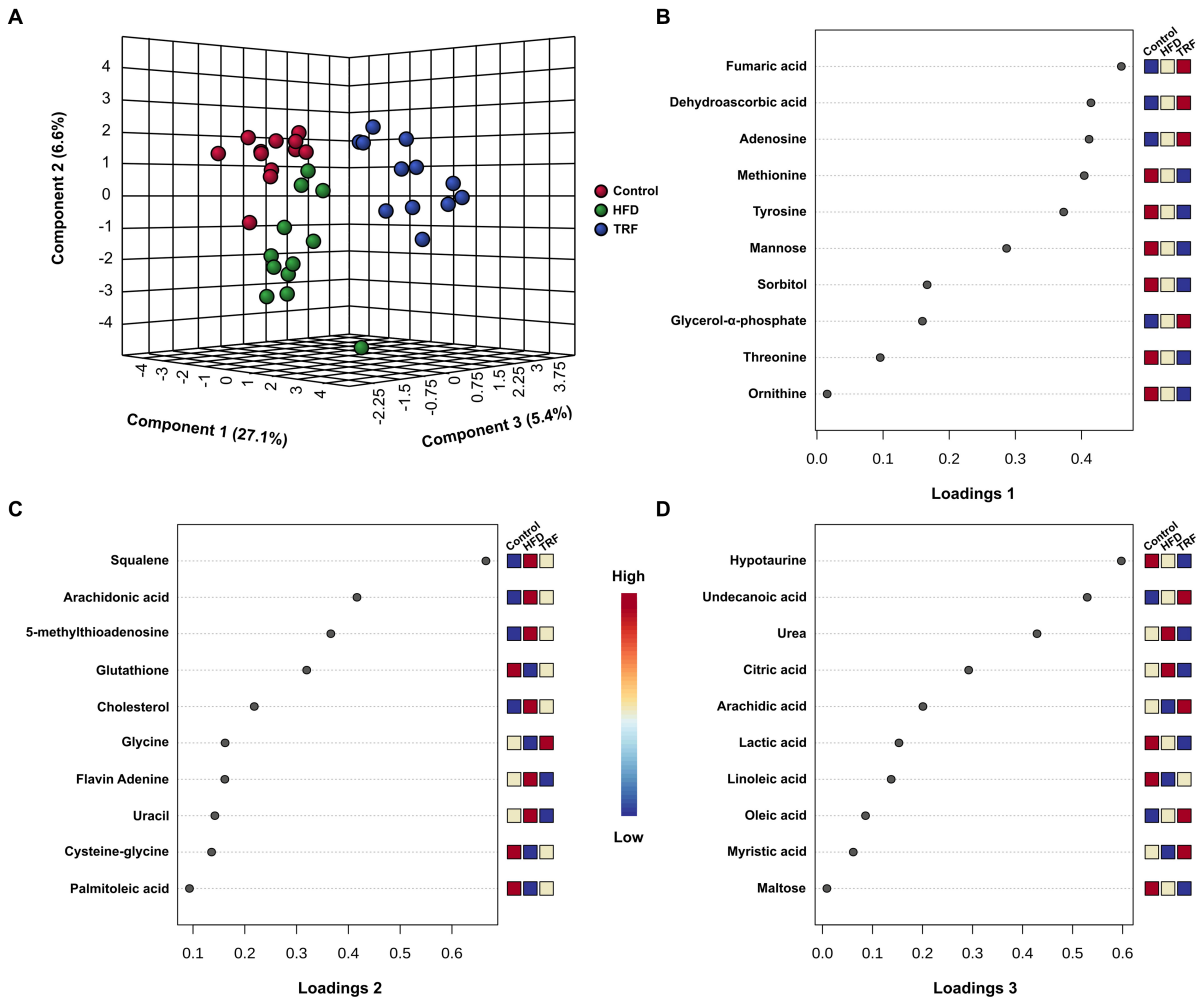
The sPLS-DA scores plot of hepatic metabolites **(A)** and loadings plots of the 10 metabolites that are the most influential in treatment separation among the control, high-fat diet (HFD), and time-restricted feeding (TRF) of the HFD groups for components one **(B)**, two **(C)**, and three **(D)** (*n* = 12 per group). The x-axis of loadings plots shows that variables are ranked by the absolute values of their loadings. The dark-red color shows greater signal strength and the blue color shows weaker signal strength by GC-TOF-MS.

The sPLS-DA scores plot exhibited separation of the three groups (Figure 5A). Along the x-axis, the TRF group was separated from the control and HFD groups in component one, which constituted 27.1% of the total separation. Along the y-axis, the HFD group tended to be separated from the control and TRF groups in component two (Figure 5A).

The 10 major determinates for separation in the loadings plot of component one were amino acid metabolites (methionine, tyrosine, threonine, and ornithine), energy metabolism metabolites (fumaric acid, mannose, sorbitol, and glycerol-α-phosphate), vitamin metabolite dehydroascorbic acid, and nucleotide adenosine (Figure 5B). The loadings plot of component two indicated that lipid metabolites (squalene, arachidonic acid, cholesterol, and palmitoleic acid), amino acid metabolites (glutathione, glycine, and cysteine-glycine), nucleotide metabolites 5-methyl-thioadenosine and uracil, and vitamin metabolite flavin adenine were the 10 major determinants for separation (Figure 5C). In the loadings plot of component three, lipid metabolites (undecanoic acid, arachidic acid, linoleic acid, oleic acid, and myristic acid), energy metabolites (citric acid, lactic acid, and maltose), and amino acid metabolites hypotaurine and urea were the 10 major determinants for separation (Figure 5D).

**Figure 6:**
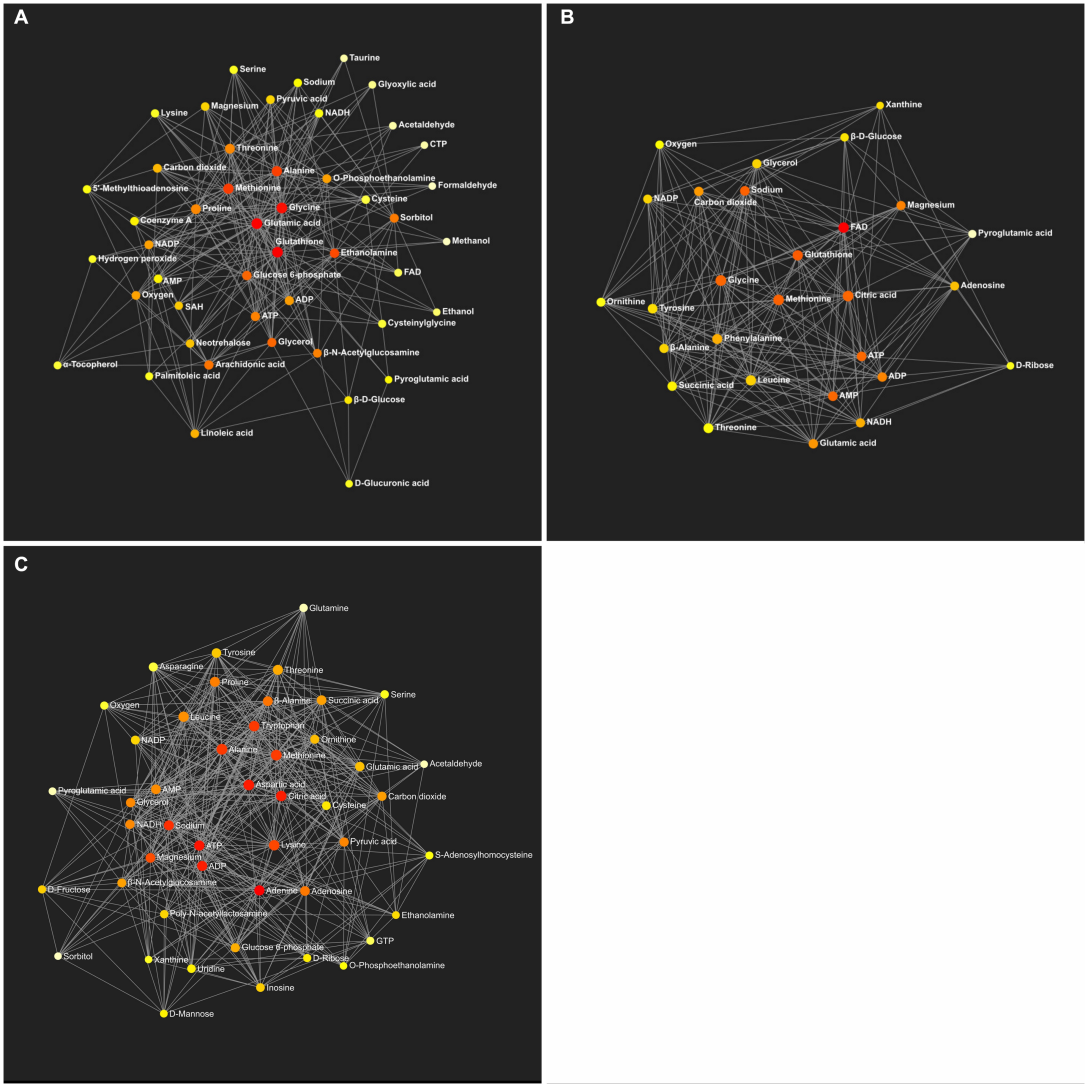
Metabolic network generated by the identified metabolites between the control and HFD **(A)**, the HFD and TRF **(B)**, and the control and TRF groups **(C)** (*n* = 12 per group). Colors from white-yellow to red represent levels of impact the metabolites have on the network in an ascending order (the number of connections a node has to other nodes and the number of shortest paths going through the node). Network statistics for **A–C** are presented in Table 8 and Supplementary Tables S3–S5, respectively.

The KEGG global metabolic network analysis identified 33 metabolic pathways when the HFD group was compared to the control group (Supplementary Table S3). Among them, the aminoacyl-tRNA biosynthesis and pathway and the glutathione metabolism pathway were altered significantly (Table 8). The analysis showed 28 metabolic pathways when the TRF group was compared to the HFD group (Supplementary Table S4). Three pathways were significantly altered; these were the aminoacyl-tRNA biosynthesis, glutathione metabolism, and phenylalanine, tyrosine, and tryptophan biosynthesis pathways (Table 8). The analysis identified 44 metabolic pathways when the TRF group was compared to the control group (Supplementary Table S5). The aminoacyl-tRNA biosynthesis pathway and the alanine, aspartate, and glutamate metabolism pathway were altered significantly (Table 8). Functional relationships that were mapped on the basis of the identified metabolites between the control and HFD, the HFD and TRF, and the control and TRF groups are presented in Figure 6.

**Table 8:** Network enrichment pathways identified by the KEGG global metabolic network analysis that are significantly altered by the high-fat diet or time-restricted feeding of the high-fat diet.

Metabolic pathways	Match status^a^	*p* ^b^
Control vs high-fat diet		
Aminoacyl-tRNA biosynthesis	6/22	< 0.01
Glutathione metabolism	4/19	< 0.01
High-fat diet vs time-restricted feeding		
Aminoacyl-tRNA biosynthesis	7/22	< 0.01
Glutathione metabolism	4/19	< 0.01
Phenylalanine, tyrosine, and tryptophan biosynthesis	2/4	0.04
Control vs time-restricted feeding		
Aminoacyl-tRNA biosynthesis	10/22	< 0.01
Alanine, aspartate, and glutamate metabolism	6/28	< 0.01

a Number of identified metabolites that match to pathway metabolites.

b FDR-adjusted *p* values.

## Discussion

In the present study, TRF in the dark phase mitigated metabolic disturbance and restored the impaired metabolic flexibility in adult mice with excess adiposity. As indicated in the following discussion, the restoration of impaired metabolic flexibility or the ability to effectively oxidize carbohydrate in the fed condition and change to burning fat in the fasted condition was attested by reduced weight gain, decreased obesity-related metabolic biomarkers (e.g., insulin, leptin, and total cholesterol in plasma and triacylglycerol in liver), increased adiponectin, and reestablishment of diurnal oscillation of RER by TRF.

RER is an indicator of which fuel is being utilized to supply energy to the body, with a ratio of 1 for carbohydrates and 0.7 for the most common fatty acids (30). In metabolically healthy rodents, RER oscillates within the range from 0.7 to 1 on a daily basis, with a lower RER in the light phase and a greater RER in the dark phase. Our finding of diminished metabolic flexibility in HFD-fed mice is consistent with finding in humans with obesity and type 2 diabetes (31) and in rodents consuming a high-fat diet (10, 11, 32). In this study, the ability of TRF to reassert RER oscillation so that fat oxidation occurs in the light phase and carbohydrate oxidation occurs in the dark phase is evidence for the restoration of metabolic flexibility in mice with excess adiposity. The restoration of metabolic flexibility and metabolic normality improvement in TRF mice is emphasized by the improvement in insulin sensitivity and changes in intermediate metabolic signals of both gluconeogenesis (e.g., glycerol-α-phosphate) and the citrate cycle (e.g., citric acid and succinic acid). These changes suggest that TRF may increase energy expenditure in adult mice with excess body fat mass.

Improvement in metabolic normality by TRF was also reflected by changes in several cytokine and lipid variables in plasma and liver. Plasma leptin, adiponectin, total cholesterol, HDL cholesterol, and hepatic triacylglycerol in TRF mice more closely resembled control mice than HFD-fed mice. The significant elevation of plasma adiponectin in TRF mice, compared to the HFD-fed mice, suggests that metabolic normality apparently was restored in TRF mice, although they continued consuming the HFD and had excess adiposity.

Increased signals of adenosine and succinic acid and decreased signal of flavin adenine found in TRF mice are consistent with the restoration of abnormal RER oscillations to normal in TRF mice. Adenosine is a building block for adenosine triphosphate (ATP). Flavin adenine dinucleotide (FAD) is a hydrogen carrier derived from the vitamin riboflavin. In the citrate cycle, FAD is reduced to FADH_2_ during the succinate dehydrogenase-catalyzed conversion of succinate to fumarate. Changes found in the present study indicate a more normally functioning citrate cycle in TRF mice than in HFD-fed mice because they have been prompted to oxidize body fat diurnally when energy from diet is unavailable.

TRF apparently altered amino acid metabolism. The aminoacyl-tRNA biosynthesis pathway was altered significantly when the TRF group was compared to either the HFD or control group. Aminoacyl-tRNA synthetases are a family of enzymes that play a critical role in protein synthesis (33). It includes both the phenylalanine, tyrosine, and tryptophan biosynthesis pathway and the alanine, aspartate, and glutamate metabolism pathway that were altered by TRF. Decreased signals of phenylalanine and tyrosine in TRF mice further support the notion that TRF alters amino acid metabolism. Both phenylalanine and tyrosine are major constituents in the phenylalanine, tyrosine, and tryptophan biosynthesis pathway.

Alteration of the glutathione metabolism pathway occurred in TRF mice. Glutathione (an antioxidant) is a tripeptide composed of glutamate, cysteine, and glycine. In glutathione metabolism, glutamate is removed from glutathione by γ-glutamyltranspeptidase to produce cysteine-glycine (34). Elevated serum concentrations of γ-glutamyltranspeptidase occur in humans with obesity-related morbidities (35). Downregulation of glutathione occurs in mice fed a HFD (36). Furthermore, γ-glutamyltranspeptidase produces cysteine-glycine to enhance antioxidant capacity (37). In the present study, decreases in cysteine-glycine signal occurred in both HFD-fed and TRF mice, and glutathione signals were unaffected in both groups. These findings suggest an altered γ-glutamyltranspeptidase activity due to an increased demand for antioxidant activity in mice-fed the HFD regardless of TRF or not. The findings also indicate a perturbation of glutathione metabolism and a diminished antioxidant capacity in HFD-fed mice with excess adiposity that is not overcome by TRF.

The increased adenosine signal and decreased ribose and xanthine signals as a result of TRF indicate that TRF alters nucleotide metabolism. Adenosine is a component of newly synthesized RNA for protein translation. Adenosine consists of an adenine (a purine) attached to a ribose. Xanthine is a breakdown product in purine degradation. Our results suggest that in TRF mice, adenosine formation may have increased, purine breakdown decreased, or both.

A HFD alters circadian rhythms that results in metabolic disturbance in laboratory rodents (11–13, 19, 38). The *Per* family is one of the core circadian elements that plays important roles in maintaining diurnal rhythms of biological clocks and normal metabolic function. Elevated *PER2* occurs in humans with abdominal obesity (39). PER2 deficiency alters lipid metabolism with reductions in total triacylglycerol and non-esterified fatty acids (40). Disturbance of diurnal feeding rhythms occurred in *Per1* mutant (41) and *Per2* knockout mice (42); these mice gained excess fat mass when they were fed a HFD. In the present study, TRF mitigated the HFD-elevated transcription of both *Per1* and *Per 2* to the levels observed in control mice. This mitigation may have contributed, at least partly, to the TRF-mediated metabolic restoration in HFD-fed mice.

Findings were obtained that TRF might have beneficial effects in obesity through modulating hepatic lipid metabolism. Transcription of *Acaca*, *Fads1*, *Fads2*, *Fasn*, *Scd1*, and *Srebf1* were diminished in TRF mice. This likely was a result of entrainment of circadian rhythms by TRF that attenuates the transcription of these genes in the light phase under the fasting condition when lipid oxidation may prevail. Thus, TRF may have reduced hepatic biosynthesis of nonessential fatty acids. This suggestion is supported by the finding that TRF lowered plasma insulin in HFD-fed mice. Insulin upregulates *Srebf1* in rat hepatocytes by activating mammalian target of rapamycin complex 1 (mTORC1) (43). SREBF1 induces lipogenesis and facilitates the storage of fatty acids in the form of triacylglycerol in liver (44). Altering this process such that hepatic triacylglycerol is decreased supports the notion that TRF mitigates nonessential fatty acid biosynthesis in adult mice with excess adiposity.

In the present study, hepatic squalene was lower in TRF mice than in HFD-fed mice. Squalene is an early intermediate in sterol biosynthesis, including cholesterol and steroid hormones (45, 46). The decreased squalene signal is consistent with the decreased total cholesterol in plasma of TRF mice. These findings are supported by previous reports that TRF reduces blood cholesterol concentrations in mice fed a HFD (11, 32). The present findings suggest that TRF may suppress cholesterol biosynthesis in its early stages.

In this study, we found that TRF did not decrease body fat mass in mice with excess adiposity. This finding contrasts with previous reports that TRF of a HFD prior to induction of excess adiposity decreases body fat mass in mice (10, 11, 19) and with clinical trials showing that time-restricted eating decreases body mass index (17) and visceral fat mass (18) in overweight and obese human subjects. Possible explanations for this discrepancy may be differences between animal studies and human trials and differences in study designs of animal studies (e.g., the status of fatness at which the TRF is initiated and the number of hours of restricted feeding applied to mice).

In a previous study, it was found that fasting more than 12 h per day (e.g., an 8-h TRF with a 16-h fasting) reduces energy intake such that growth may be retarded in mice (10). In the present study, there were no differences in energy intake among the three groups, nor was there a significant difference in body weight over the course of study when TRF mice were compared to control mice. Thus, the metabolomic alteration observed in TRF mice is a result of TRF rather than a result of reduced nutrient intake or growth retardation.

In conclusion, TRF improved metabolic function in adult mice with excess adiposity and created a metabolomic signature in liver distinct from both control and HFD-fed mice. This improvement was not through a reduction in body fat mass but rather through the restoration of metabolic flexibility. Findings from this study suggest that restriction of mealtime to the active phase of the day may improve metabolic health in subjects who are overweight or obese.

## Data availability statement

The original contributions presented in the study are included in the article/Supplementary material, further inquiries can be directed to the corresponding author.

## Ethics statement

The animal study was approved by the Institutional Animal Care and Use Committee of the Grand Forks Human Nutrition Research Center, Agricultural Research Service, USDA. The study was conducted in accordance with the local legislation and institutional requirements.

## Author contributions

LY: Conceptualization, Formal analysis, Funding acquisition, Investigation, Methodology, Validation, Writing – original draft, Writing – review & editing. BR: Formal analysis, Validation, Writing – original draft, Writing – review & editing. DP: Formal analysis, Validation, Writing – original draft, Writing – review & editing.
